# Supplementary material for: Improving early epidemiological assessment of emerging Aedes-transmitted epidemics using historical data
Source: PLoS Negl Trop Dis. 2018 Jun 4;12(6):e0006526. doi: 10.1371/journal.pntd.0006526 (PMC6002135; doi:10.1371/journal.pntd.0006526)
Supplement: S1 Appendix — (PDF) [file pntd.0006526.s001.pdf]

# Supplementary Information: Improving early epidemiological assessment of emerging *Aedes*-transmitted epidemics using historical data

Julien Riou[a,b,*], Chiara Poletto[a], and Pierre-Yves Boëlle[a]

[a]Sorbonne Université, INSERM, Institut Pierre Louis d'épidémiologie et de Santé Publique, IPLESP UMR-S1136, F-75012 Paris, France
[b]EHESP School of Public Health, Rennes, France
[*]Corresponding author: julien.riou@iplesp.upmc.fr

## Contents

**1 Models** **2**
1.1 One island, one disease: dataset $\mathcal{D}1$ . . . 2
1.2 Several islands, one disease: dataset $\mathcal{D}2$ . . . 3
1.3 Several islands, two diseases: dataset $\mathcal{D}3$ . . . 3

**2 Serial interval** **5**

**3 Information extracted from historical data** **7**

**4 Parameter estimates for the ZIKV epidemics in the French West Indies** **8**

**5 Sensitivity analyses and comparison with alternative priors** **9**
5.1 Impact of the serial interval . . . 9
5.2 Using information from CHIKV in the same island without adjusting for the differences between CHIKV and ZIKV . . . 10
5.3 Using information from the ZIKV epidemics in other islands . . . 11
5.4 The respective impact of the informative priors on $\rho$ and $\mathcal{R}_0$ . . . 12
5.5 Informative priors on the overdispersion parameter . . . 13

**6 Stan code** **14**
6.1 Model for $\mathcal{D}1$ . . . 14
6.2 Model for $\mathcal{D}2$ . . . 16
6.3 Model for $\mathcal{D}3$ . . . 18

**7 References** **21**

# 1 Models

Here, we provide a detailed description of three different models adopted for the reconstruction of the informative priors and for providing forecast on the ZIKV epidemics in French West Indies. A *one island, one disease* model was adopted to forecast ZIKV epidemics in each island of French West Indies based on dataset $\mathcal{D}1$ and the three different prior choices presented in the main paper. A *several islands, one disease* approach was used for modelling jointly several CHIKV epidemics in the three islands of the French West Indies (based on dataset $\mathcal{D}2$), and obtaining the posteriors on regional and local CHIKV parameters as described in the first step of the procedure presented in the main paper. A *several islands, several disease* model [?] was used for the several CHIKV and ZIKV epidemics in six islands of French Polynesia (dataset $\mathcal{D}3$) to recover the ratio between ZIKV and CHIKV parameters as described in the second step in the main paper.

## 1.1 One island, one disease: dataset $\mathcal{D}1$

We are interested in modelling observed ZIKV incidence data in an island of the French West Indies available (up to week $K$) and producing forecasts of future observed incidence (up to week $K+104$). We consider the time series $O = \{O_t\}_{t=1,\cdots,K}$ of the weekly number of incident cases reported to the surveillance system. $O$ consists of the cases who sought clinical advice and were diagnosed, a fraction of the (unobserved) incident infected cases $I = \{I_t\}_{t=1,\cdots,K}$. We therefore write, in the "observation" level of the model, that $O_t$ is a proportion $\rho$ of all cases $I_t$ according to:

$$O_t | I_t, \rho \sim \text{Binom}(I_t, \rho), \tag{1}$$

where $\rho$ is the probability that an infected case consulted with a health professional, was diagnosed and reported, thereafter refered to as the reporting rate. In the "transmission" level, we link incidence $I_t$ with past observed incidences $O_t^- = \{O_1, \cdots, O_{t-1}\}$ as :

$$I_t | O_t^-, \mathcal{R}_0, \rho \sim \text{Binom}\left(S_t, \frac{\mathcal{R}_0}{N} \sum_{n=1}^{5} w_{t,n} \frac{O_{t-n}}{\rho}\right), \tag{2}$$

where $N$ the total population of the island and $\mathcal{R}_0$ is a transmission parameter. The term $\sum_{n=1}^{5} w_{t,n} O_{t-n}/\rho$ summarizes exposure to infectious mosquitoes at time $t$: it is an average of past incidence with weights defined by the serial interval distribution $w_{t,n} = G(n+0.5; \bar{T}_t) - G(n-0.5; \bar{T}_t)$, as discussed in section 2 of the supplementary appendix. The number of susceptible individuals $S_t = N - \sum_{u=1}^{t-1} I_u$ at the beginning of period $t$ is computed as $N - \sum_{u=1}^{t-1} O_u/\rho$, noting that $O_u/\rho$ is a first order approximation to $I_u$. To avoid data augmentation with the unobserved $I$ during estimation, we collapse the "observation" and "transmission" levels into a single binomial distribution:

$$O_t | O_t^-, \rho, \mathcal{R}_0 \sim \text{Binom}\left(S_t, \frac{\mathcal{R}_0}{N} \sum_{n=1}^{5} w_{t,n} O_{t-n}\right). \tag{3}$$

In a final step, we account for the imprecise nature of the $O$ data, since observed cases $O$ have been extrapolated from limited information provided by a network of local health practitioners. We therefore allow for over-dispersion using a negative binomial distribution instead of the binomial, as:

$$O_t | O_t^-, \rho, \mathcal{R}_0, \phi \sim \text{Neg-Binom}\left(S_t \frac{\mathcal{R}_0}{N} \sum_{n=1}^{5} w_{t,n} O_{t-n}, \phi\right), \tag{4}$$

where variance is computed as the mean divided by $\phi$. The joint probability of data and parameters is finally

$$\pi(O, \rho, \mathcal{R}_0, \phi) = \left\{\prod_t \pi(O_t | O_t^-, \rho, \mathcal{R}_0, \phi)\right\} \pi(\rho)\pi(\mathcal{R}_0)\pi(\phi), \tag{5}$$

where $\pi(\mathcal{R}_0)$, $\pi(\rho)$ and $\pi(\phi)$ are prior distributions, which could either be non-informative or informative (Table 1), the implications of this choice being the main interest of the paper. The forecasts of future incidence rely upon a stochastic model following equation (4), and are obtained using the full posterior distributions of $\mathcal{R}_0$, $\rho$ and $\phi$.

## 1.2 Several islands, one disease: dataset $\mathcal{D}2$

Here, we aim to jointly model the three CHIKV epidemics in Martinique, Guadeloupe and Saint-Martin, with the objective of extracting information from these past epidemics and use it to improve forecasting. We introduce a hierarchical structure in the model for reporting rates and transmission, with island levels nested within a regional level. Reporting rates $\rho_i$ in island $i$ are modelled using a logistic-normal model

$$\ln \frac{\rho_i}{1-\rho_i} = r_i \tag{6}$$

where $r_i \sim \mathcal{N}(\mu_\rho, \sigma_\rho^2)$ is a random island-specific effect. Reporting is thus controlled at the regional levels by two hyperparameters, $\theta_{rho} = \{\mu_\rho, \sigma_\rho\}$. Likewise, we allow for a random island-specific coefficient in the transmission term, with

$$\mathcal{R}_{0i} \sim \mathcal{N}(\mu_{\mathcal{R}_0}, \sigma_{\mathcal{R}_0}^2) \tag{7}$$

Transmission is thus controlled at the regional level by two hyperparameters $\theta_{\mathcal{R}_0} = \{\mu_{\mathcal{R}_0}, \sigma_{\mathcal{R}_0}\}$. Writing $O_i$ the observed incidence in the outbreak in island $i$, and $O$ the whole dataset, we have:

$$\pi(O, \theta_\rho, \theta_{\mathcal{R}_0}, \phi) = \left\{ \prod_i \pi(O_i | O_i^-, \mathcal{R}_{0i}, \rho_i, \phi) \pi(\rho_i|\theta_\rho) \pi(\mathcal{R}_{0i}|\theta_{\mathcal{R}_0}) \right\} \pi(\theta_\rho) \pi(\theta_{\mathcal{R}_0}) \pi(\phi) \tag{8}$$

We chose weakly informative prior distributions for all parameters (Table A).

| Parameter | Prior distribution | Comments |
|---|---|---|
| $\mu_\rho$ | $\mathcal{N}(0, 1.5^2)$ | Implies a uniform distribution between 0 and 1 on after inverse-logit transformation. |
| $\sigma_\rho$ | Cauchy$(0, 2.5)$ | Weakly informative prior for variance parameters in [?]. |
| $\mu_{\mathcal{R}_0}$ | Gamma$(1, 0.2)$ | Implies that $\mathcal{R}_0$ is stricly positive, away from 0 and probably between 1 and 5. |
| $\sigma_{\mathcal{R}_0}$ | Cauchy$(0, 2.5)$ | Weakly informative prior for variance parameters in [?]. |
| $\phi$ | Cauchy$(0, 2.5)$ | Weakly informative prior for variance parameters in [?]. |

Table A: Prior distributions used for modelling dataset $\mathcal{D}2$.

We thus obtained posterior estimates of the epidemiological parameters of CHIKV epidemics in the French West Indies, from which were derived the informative priors used when forecasting ZIKV in the same area (local priors in island $i$ being derived from $\pi(\mathcal{R}_{0i}|\mathcal{D}2)$ and $\pi(\rho_i|\mathcal{D}2)$ and regional priors from $\pi(\mu_{\mathcal{R}_0}|\mathcal{D}2)$, $\pi(\sigma_{\mathcal{R}_0}|\mathcal{D}2)$, $\pi(\mu_\rho|\mathcal{D}2)$ and $\pi(\sigma_\rho|\mathcal{D}2)$).

## 1.3 Several islands, two diseases: dataset $\mathcal{D}3$

Last, we jointly model the successive epidemics of CHIKV and ZIKV in six islands or archipelagoes of French Polynesia. Building from the hierarchical model introduced in section (1.2), we add a fixed effect for the disease. Reporting rates $\rho_{ij}$ in island $i$ for disease $j$ are therefore modelled using a logistic-normal model

$$\ln \frac{\rho_{ij}}{1-\rho_{ij}} = r_i + V_j \ln \beta_\rho \tag{9}$$

where $r_i \sim N(\mu_\rho, \sigma_\rho^2)$ is an island-specific random parameter corresponding to the reporting rate of CHIKV in island $i$, $V_j$ is 1 for ZIKV and 0 for CHIKV and $\beta_\rho$ is the ratio of reporting ZIKV cases relative to CHIKV cases during an epidemic. Three parameters $\theta_\rho = \{\mu_\rho, \sigma_\rho, \beta_\rho\}$ thus control reporting. Likewise, transmission is modelled as follows:

$$\ln \mathcal{R}_{0ij} = \ln b_i + V_j \ln \beta_{\mathcal{R}_0} \tag{10}$$

where $b_i \sim \mathcal{N}(\mu_{\mathcal{R}_0}, \sigma_{\mathcal{R}_0}^2)$ is an island-specific random parameter corresponding to the transmission of CHIKV in island $i$, $V_j$ is 1 for ZIKV and 0 for CHIKV and $\beta_{\mathcal{R}_0}$ is the relative transmission of ZIKV compared

to CHIKV. Transmission thus depends on parameters $\theta_{\mathcal{R}_0} = \{\mu_{\mathcal{R}_0}, \sigma_{\mathcal{R}_0}, \beta_{\mathcal{R}_0}\}$. Writing $O_{ij}$ the observed incidence in the outbreak in island $i$ and disease $j$, and $O$ the whole dataset, we have:

$$\pi(O, \theta_\rho, \theta_{\mathcal{R}_0}, \phi) = \left\{ \prod_{i,j} \pi(O_{ij}|O_{ij}^{-}, \mathcal{R}_{0ij}, \rho_{ij}, \phi)\pi(\rho_{ij}|\theta_\rho)\pi(\mathcal{R}_{0ij}|\theta_{\mathcal{R}_0}) \right\} \pi(\theta_\rho)\pi(\theta_{\mathcal{R}_0})\pi(\phi) \tag{11}$$

There again, we chose weakly informative prior distributions for all parameters (Table B).

| Parameter | Prior distribution | Comments |
|---|---|---|
| $\mu_\rho$ | $\mathcal{N}(0, 1.5^2)$ | Implies a uniform distribution between 0 and 1 after inverse-logit transformation. |
| $\sigma_\rho$ | Cauchy$(0, 2.5)$ | Weakly informative prior for variance parameters in [?]. |
| $\beta_\rho$ | Exp$(1)$ | Weakly informative prior adapted to ratios. |
| $\mu_{\mathcal{R}_0}$ | Gamma$(1, 0.2)$ | Implies that $\mathcal{R}_0$ is stricly positive, away from 0 and probably between 1 and 5. |
| $\sigma_{\mathcal{R}_0}$ | Cauchy$(0, 2.5)$ | Weakly informative prior for variance parameters in [?]. |
| $\beta_{\mathcal{R}_0}$ | Exp$(1)$ | Weakly informative prior adapted to ratios. |
| $\phi$ | Cauchy$(0, 2.5)$ | Weakly informative prior for variance parameters in [?]. |

Table B: Prior distributions used for modelling dataset $\mathcal{D}3$.

We thus obtained posterior estimates of the difference between reporting and transmission during CHIKV and ZIKV epidemics occuring in the same locations, that is $\pi(\beta_\rho|\mathcal{D}3)$ and $\pi(\beta_{\mathcal{R}_0}|\mathcal{D}3)$, respectively. These were combined with the information extracted from dataset $\mathcal{D}2$ to build the local and regional informative priors.

## 2 Serial interval

Here we provide detailed description of the reconstruction of the serial interval distribution. We used the framework that we previously developed [?, ?, ?]. It describes the different stages of disease progress in the infected humans and vectors from the development of symptoms in the primary case to development of symptoms in the secondary case. Precisely, we split the distribution of the serial interval $T_{SI}$ in four components:

1. Time from infectiousness to symptoms in a human case $T_V$, distributed as:

$$T_V(t|\tau_V) = \mathcal{U}(0, \tau_V), \tag{12}$$

where $\tau_V$ is the maximum duration from infectiousness to symptoms.

2. Time from infectiousness to infectious mosquito bite $T_B$, such as:

$$T_B(t|\tau_a, \tau_b) = \mathcal{U}[0, \mathcal{U}(\tau_a, \tau_b)], \tag{13}$$

where $\tau_a$ and $\tau_b$ are the minimum and maximum durations of the infectious period, respectively.

3. Time from the bite infecting the mosquito to the bite transmitting the disease $T_M$, which depends on the extrinsic incubation period and is a multiple of the duration of the gonotrophic cycle of the mosquito. The mosquito bite can transmit the virus once the extrinsic incubation period in the mosquito is complete. Then, every bite, occurring at regular intervals around the average duration of one gonotrophic cycle ($\pm 1/3$), can lead to transmission. This quantity is modelled as as mixture of time to bite $T_M = \sum_i T_{M,c} 1\{C = i\}$ , with $T_{M,c}$ the duration from initial mosquito infection to a bite in the $c$-th gonotrophic cycle, with distribution :

$$T_{M,c}(t|\gamma, \kappa) = \mathcal{U}(\kappa + (c - 1/3)\gamma, \kappa + (c + 1/3)\gamma), \tag{14}$$

where $\gamma$ is the average duration of a gonotrophic cycle, $\kappa$ is the duration of the extrinsic incubation period. The weights of each $T_{M,c}$ component is proportional to $e^{-\delta c \gamma}$ where $\delta$ is the mosquito mortality: this corresponds with the fraction of mosquitoes surviving long enough to bite during the $c$-th cycle. Depending on the gonotrophic cycle duration, the number of components in the mixture changes.

4. The incubation period (from infection to disease onset) in a secondary case $T_I$, which follows a lognormal distribution of mean $\mu_I$ and standard deviation $\sigma_I$.

Using these components, the serial interval is:

$$T_{SI} = -T_V + T_B + T_M + T_I \tag{15}$$

According to this formulation, the distribution of $T_{SI}$ depends on eight parameters, five of which relate only to the human host ($\mu_I$, $\sigma_I$, $\tau_V$, $\tau_a$, and $\tau_b$), and are constant over time for a given virus (Table C). The other three parameters ($\delta$, $\gamma$, and $\kappa$) depend on the vector and as so are susceptible to vary according to temperature. This study, contrary to [?], revolved around producing forecasts of the future course of the epidemic in real time. While including meteorological predictions might bring additional improvements to forecast quality, we decided for the sake of simplicity to ignore the influence of local temperature on the serial interval and assume a constant temperature of 28°C for the whole duration of the epidemics. In the following, we made the assumption that the main vector of ZIKV and CHIKV was *Ae. aegypti*. This was decided considering that *Ae. aegypti* is a competent vector of both viruses and is widely prevalent in the concerned areas [?, ?, ?, ?]. We thus searched for published estimates of these three parameters in laboratory or preferably field studies involving *Ae. aegypti* (Table C).

| Stage | Symbol | Definition | CHIKV | ZIKV | Unit | References |
|---|---|---|---|---|---|---|
| $(i)$ | $\tau_V$ | Maximum time from infectiousness to symptoms in humans | 3 | 3 | Days | [?, ?, ?, ?] |
| $(ii)$ | $\tau_a - \tau_b$ | Range of the duration of the infectious period in humans | 3 - 8 | 4 - 7 | Days | [?, ?, ?, ?] |
| $(iii)$ | $\kappa$ | Extrinsic incubation period at 28°C | 3 | 6 | Days | [?, ?, ?] |
| $(iii)$ | $\gamma$ | Length of the gonotrophic cycle at 28°C | 3 | 3 | Days | [?] |
| $(iii)$ | $\delta$ | Daily probability of death for *Ae. aegypti* | 0.29 | 0.29 | - | [?] |
| $(iv)$ | $\mu_I$, $\sigma_I$ | Mean and standard deviation of the incubation period in humans | 3.0, 1.3 | 5.9, 1.5 | Days | [?, ?, ?, ?] |

Table C: Parameters used for the computation of the distribution of the serial interval.

This resulted in distributions for the serial interval best summarized by gamma distributions with mean 2.5 weeks (standard deviation 0.7) for ZIKV and mean 1.6 weeks (standard deviation 0.6) for CHIKV. These distributions were then discretized to be used as the weights $w_t$ in the statistical models (Fig. A).

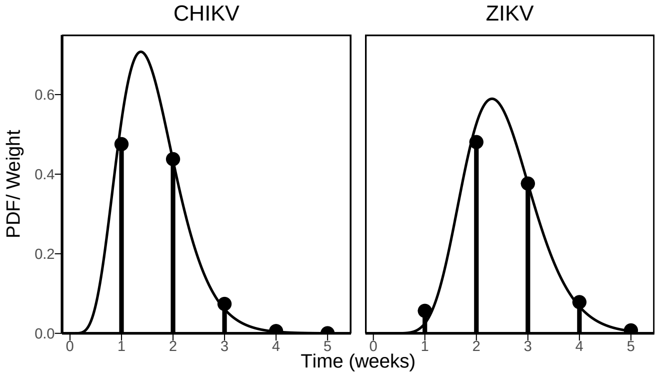


Figure A: Probability distribution of the serial interval for CHIKV and ZIKV (lines) and corresponding discrete weights to be used in the models (dots).

# 3 Information extracted from historical data

The analysis of past CHIKV outbreaks in the French West Indies (dataset $\mathcal{D}2$) using the model described in section 1.2 and of past ZIKV and CHIKV outbreak in French Polynesia (dataset $\mathcal{D}3$) using the model described in section 1.3 led to posterior distributions for $R_0$ and $\rho$ summarised in Table D. In addition, dataset $\mathcal{D}3$ was used to estimate the relative transmissibility (with a ratio $\beta_{\mathcal{R}_0}$ estimated to 1.03 with 95% credible interval 0.90-1.18) and the relative reporting (with a ratio $\beta_\rho$ estimated to 0.46, with 95% credible interval 0.42-0.50) of ZIKV with respect to CHIKV.

| Region | Virus | Island | Dataset | $\mathcal{R}_0$ | $\rho$ |
|---|---|---|---|---|---|
| French West Indies | CHIKV | Guadeloupe | D2 | 1.49 (1.38; 1.60) | 0.34 (0.31; 0.39) |
| – | – | Martinique | D2 | 1.26 (1.16; 1.37) | 0.50 (0.39; 0.69) |
| – | – | Saint-Martin | D2 | 1.31 (1.01; 1.70) | 0.35 (0.14; 0.87) |
| – | – | *Regional* | D2 | 1.35 (0.00; 2.76) | 0.42 (0.03; 0.95) |
| French Polynesia | CHIKV | Australes | D3 | 1.98 (1.61; 2.25) | 0.54 (0.42; 0.74) |
| – | – | Marquises | D3 | 2.06 (1.81; 2.37) | 0.63 (0.56; 0.72) |
| – | – | Mo'orea | D3 | 2.04 (1.76; 2.36) | 0.29 (0.26; 0.33) |
| – | – | Sous-le-vent | D3 | 2.04 (1.80; 2.31) | 0.31 (0.28; 0.36) |
| – | – | Tahiti | D3 | 2.04 (1.87; 2.22) | 0.29 (0.28; 0.31) |
| – | – | Tuamotus | D3 | 2.00 (1.69; 2.26) | 0.40 (0.35; 0.48) |
| – | – | *Regional* | D3 | 2.03 (1.61; 2.44) | 0.42 (0.09; 0.83) |
| French Polynesia | ZIKV | Australes | D3 | 2.03 (1.63; 2.36) | 0.25 (0.19; 0.34) |
| – | – | Marquises | D3 | 2.12 (1.82; 2.47) | 0.29 (0.25; 0.34) |
| – | – | Mo'orea | D3 | 2.10 (1.78; 2.47) | 0.13 (0.11; 0.16) |
| – | – | Sous-le-vent | D3 | 2.10 (1.80; 2.43) | 0.14 (0.13; 0.16) |
| – | – | Tahiti | D3 | 2.10 (1.86; 2.35) | 0.13 (0.13; 0.14) |
| – | – | Tuamotus | D3 | 2.05 (1.71; 2.37) | 0.18 (0.16; 0.22) |
| – | – | *Regional* | D3 | 2.09 (1.64; 2.52) | 0.21 (0.03; 0.59) |

Table D: Posterior distributions for $\mathcal{R}_0$ and $\rho$ obtained from historical data on CHIKV outbreaks in the French West Indies and on CHIKV and ZIKV outbreaks in French Polynesia. Distributions are summarized by their mean and 95% credible interval.

# 4 Parameter estimates for the ZIKV epidemics in the French West Indies

The evolution of the posterior distributions (mean and 95% credible intervals) of the basic reproduction number $\mathcal{R}_{0,Z}$ (panel A) and the reporting rate $\rho_Z$ (panel B) throughout the ZIKV epidemics of the French West Indies is shown in Fig. 5 of the main paper. In addition, we present summaries of the posteriors at dates "P" (peak) and "E" (end of the period of epidemic activity) in Table E.

| Island | Prior | $\mathcal{R}_{0,Z}$ | | $\rho_Z$ | |
|---|---|---|---|---|---|
| | | Date "$P$" | Date "$E$" | Date "$P$" | Date "$E$" |
| Guadeloupe | NI | 1.88 (1.62; 2.20) | 1.88 (1.66; 2.12) | 0.57 (0.12; 0.98) | 0.10 (0.09; 0.11) |
| – | R | 1.90 (1.63; 2.23) | 1.86 (1.63; 2.09) | 0.33 (0.10; 0.89) | 0.10 (0.09; 0.11) |
| – | L | 1.75 (1.56; 1.93) | 1.54 (1.39; 1.71) | 0.16 (0.14; 0.18) | 0.13 (0.11; 0.15) |
| Martinique | NI | 1.62 (1.15; 2.46) | 1.30 (1.04; 1.58) | 0.39 (0.05; 0.96) | 0.30 (0.16; 0.80) |
| – | R | 1.62 (1.20; 2.24) | 1.36 (1.12; 1.61) | 0.20 (0.06; 0.66) | 0.23 (0.15; 0.41) |
| – | L | 1.36 (1.18; 1.54) | 1.32 (1.19; 1.46) | 0.23 (0.17; 0.30) | 0.23 (0.18; 0.29) |
| Saint-Martin | NI | 1.50 (1.19; 2.00) | 1.34 (1.05; 1.66) | 0.44 (0.08; 0.97) | 0.25 (0.14; 0.71) |
| – | R | 1.57 (1.26; 1.98) | 1.38 (1.13; 1.66) | 0.22 (0.08; 0.69) | 0.20 (0.13; 0.35) |
| – | L | 1.50 (1.27; 1.77) | 1.38 (1.18; 1.61) | 0.18 (0.09; 0.34) | 0.19 (0.14; 0.29) |

Table E: Posterior distributions of $\mathcal{R}_{0,Z}$ and $\rho_Z$ at dates "P" (peak) and "E" (end of the period of epidemic activity) during the ZIKV epidemics in the French West Indies using different *a priori* distributions on the parameters: non-informative priors (NI) or informative priors based on historical data considered either at the regional (R) or the local (L) level. Distributions are summarized by their mean and 95% credible interval.

# 5 Sensitivity analyses and comparison with alternative priors

## 5.1 Impact of the serial interval

Our estimate of the distribution of the serial interval was based on a mechanistic reconstruction based on several assumptions (section 2). In this sensitivity analysis, we verify whether the main results would hold if the mean of the distribution of the serial interval was translated by +1 week or -1 week (Fig. B). The advantages of using informative priors, in particular local, over non-informative priors were still noticeable with this alternative parameterizations (Fig. C).

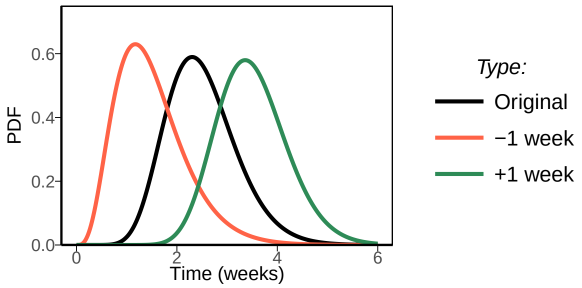


Figure B: Original and alternative distributions of the serial interval used in sensitivity analyses.

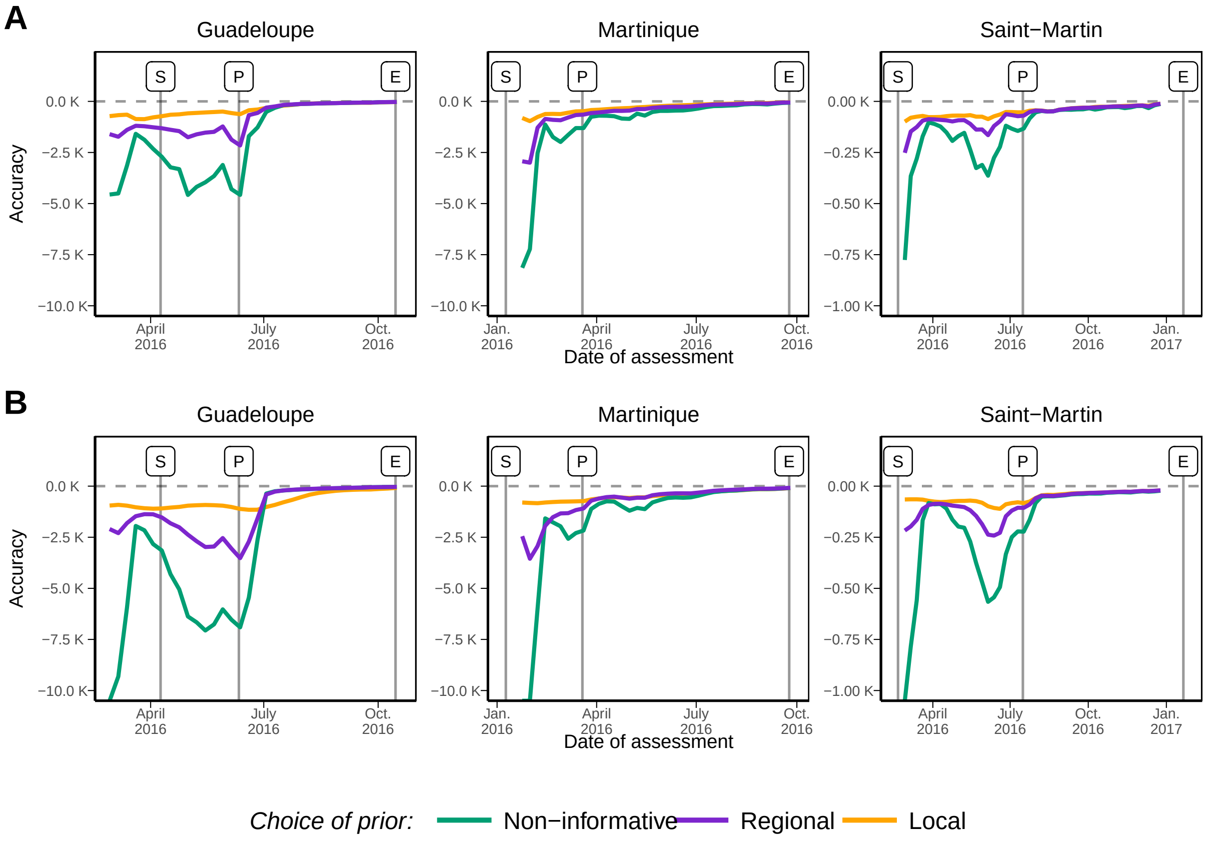


Figure C: Accuracy of the predictive distribution of future incidence based on epidemiological assessments conducted each week with the serial interval shifted by -1 week (panel A) or +1 week (panel B). Lines refer to different *a priori* distributions.

## 5.2 Using information from CHIKV in the same island without adjusting for the differences between CHIKV and ZIKV

In this sensitivity analysis, we replace the informative prior distributions on $\mathcal{R}_0$ and $\rho$ by the posterior estimates of these parameters obtained using data on CHIKV in the same islands, without adjusting for the ratios between CHIKV and ZIKV as advocated in our approach. Compared to the local priors used in the main analysis, this change mainly affects the prior on $\rho$, which was estimated in French Polynesia to be lower for ZIKV than for CHIKV ($\beta_\rho$ was estimated to 0.46 with 95% credible interval 0.42-0.50), while the estimates of $\mathcal{R}_0$ were similar for both diseases ($\beta_{\mathcal{R}_0}$ was estimated to 1.03 with 95% credible interval 0.90-1.18). The results show that omitting the ratios between ZIKV and CHIKV consistently leads to significantly lower forecasting accuracy (Fig. D). This highlights the importance of the comparative analysis between the different diseases of interest to inform the use of historical data from one disease in the epidemic forecast of the other.

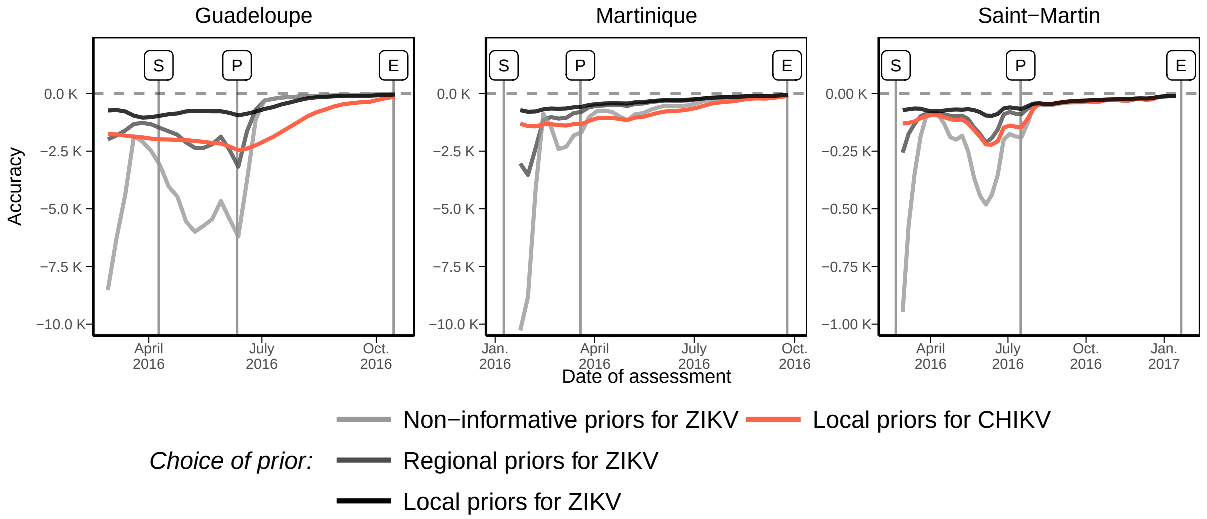


Figure D: Accuracy of the predictive distribution of future incidence based on epidemiological assessments conducted each week. Lines refer to different *a priori* distributions. The results of the main paper are shown in black and grey as a reference (see Fig. 4 of the main paper), and coloured lines correspond to alternatives tested in sensitivity analyses.

### 5.3 Using information from the ZIKV epidemics in other islands

Here, we use the posterior estimates of $\mathcal{R}_0$ and $\rho$ obtained from the ZIKV epidemics in each of six islands of French Polynesia as priors for the ZIKV epidemics in the French West Indies. Compared to the local priors built for Guadeloupe, Martinique and Saint-Martin using data from these islands, this change leads to consistently higher priors on $\mathcal{R}_0$, centered around 2 (Fig. **??**). The modifications induced on the priors on $\rho$ differ for each island. For instance, the estimates of $\rho$ in Mo'orea, the Sous-le-vent islands and Tahiti – concentrated around 0.15 – are very close to the local prior on $\rho$ applied to Guadeloupe, while they are higher in the Australes, Marquises and Tuamotu islands. These differences in the range of prior specification translate into differences in forecasting accuracy (Fig. E), with a large drop in accuracy when using estimates from the Australes, Marquises or Tuamotu islands as priors. In Martinique and Saint-Martin, local priors computed according to our approach led to better results than priors directly taken from any area of French Polynesia at the early stages. In Guadeloupe however, the accuracy obtained with priors from Sous-le-vent, Tahiti or Mo'orea was similar to that obtained with local priors computed according to our approach. This sensitivity analysis emphasizes that directly using the estimates obtained from an epidemic of the same pathogen in a different location might lead to prior misspecifications causing large inaccuracies in some cases.

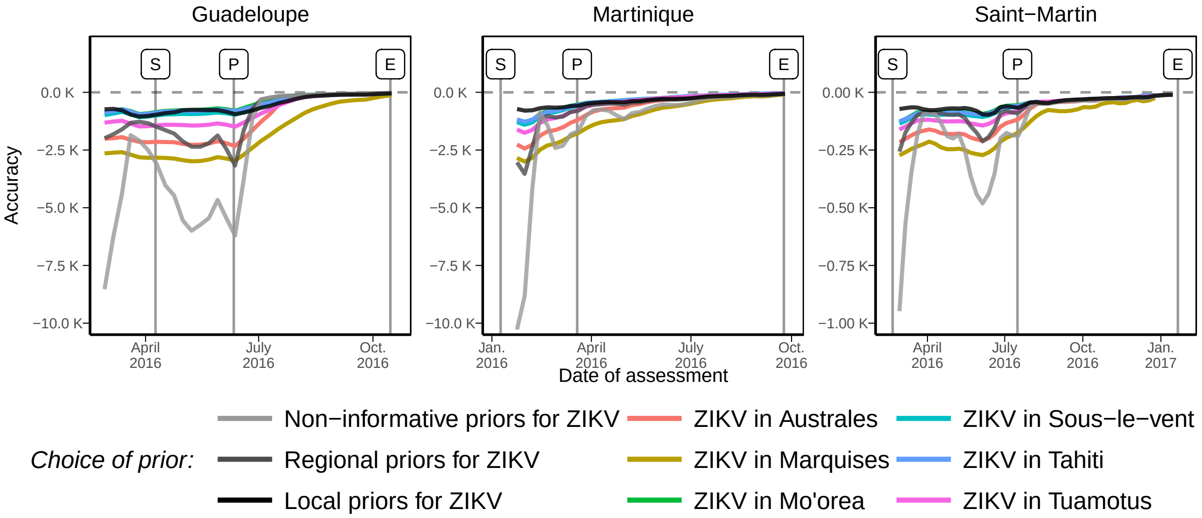


Figure E: Accuracy of the predictive distribution of future incidence based on epidemiological assessments conducted each week. Lines refer to different *a priori* distributions. The results of the main paper are shown in black and grey as a reference (see Fig. 4 of the main paper), and coloured lines correspond to alternatives tested in sensitivity analyses.

### 5.4 The respective impact of the informative priors on $\rho$ and $\mathcal{R}_0$

One of the conclusions of this work is that improvements in forecasting quality come together with improvements in the estimation of the reporting rate $\rho$ rather than of the transmission rate $\mathcal{R}_0$. This suggests that prior information is essentially required for the reporting rate, a difficult-to-estimate quantity as already noted in [**?**]. In a sensitivity analysis, we test this hypothesis, by testing separately the informative priors given on $\mathcal{R}_0$ and on $\rho$. The results confirm the greater importance of using informative priors on $\rho$ in forecasting accuracy (Fig. F). Indeed, after the first 2-4 weeks of circulation, by providing a prior for $\rho$ only we obtain the same accuracy as providing priors for $\rho$ and $\mathcal{R}_0$. This small delay is coherent with Fig. 5 of the main paper, which shows that $\mathcal{R}_0$ estimates are close to the final ones from weeks 2-4.

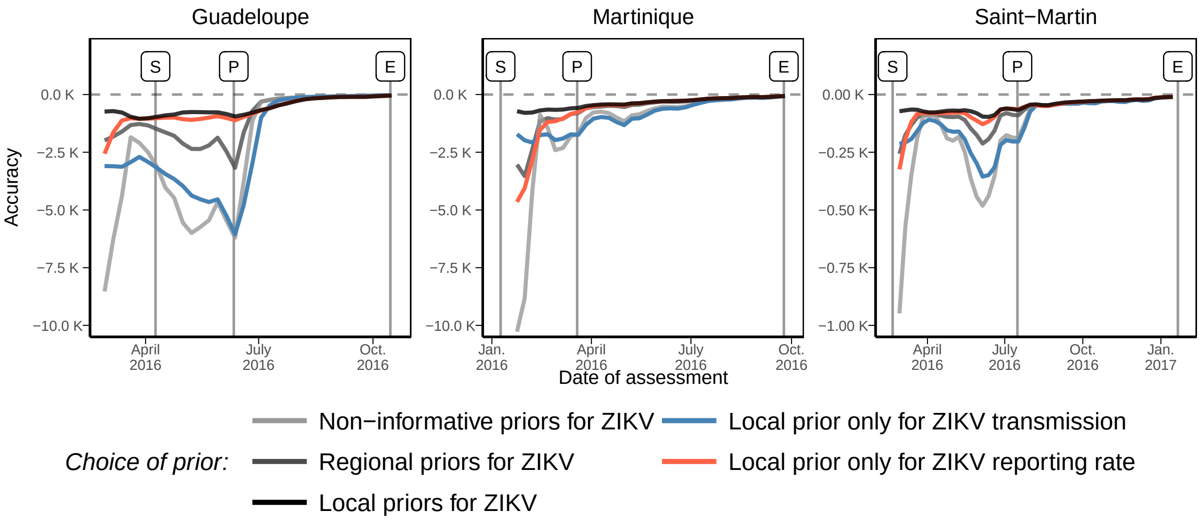


Figure F: Accuracy of the predictive distribution of future incidence based on epidemiological assessments conducted each week. Lines refer to different *a priori* distributions. The results of the main paper are shown in black and grey as a reference (see Fig. 4 of the main paper), and coloured lines correspond to alternatives tested in sensitivity analyses.

## 5.5 Informative priors on the overdispersion parameter

The parameter $\phi$ is related to the imprecise nature of observed incidence data, which was extrapolated from the limited information provided by a network of local health practitioners (see section 1.1). In this sensitivity analysis, we measure the impact of introducing an informative prior on $\phi$, obtained directly from the analysis of past CHIKV epidemics in the French West Indies. No hierarchical structure was introduced for this parameter, which was thus considered the same in every island. Moreover, we did not consider meaningful to adjust the estimate of $\phi$ obtain during the CHIKV epidemics in the French West for ZIKV, and this directly used this estimate as prior for the ZIKV epidemics in the region. Adding an informative prior on this parameter has a very limited effect on forecasting accuracy (Fig. G).

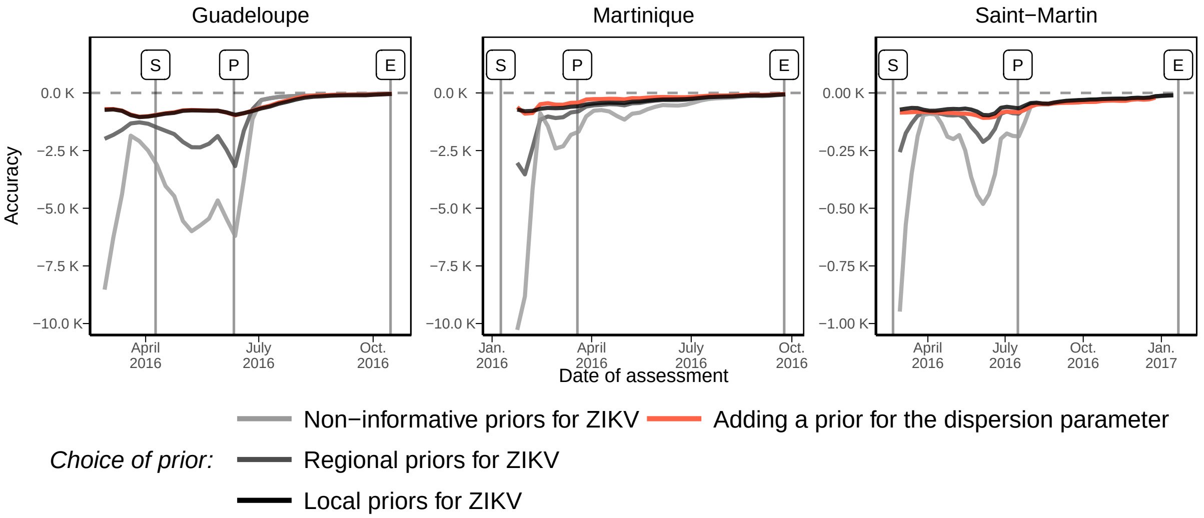


Figure G: Accuracy of the predictive distribution of future incidence based on epidemiological assessments conducted each week. Lines refer to different *a priori* distributions. The results of the main paper are shown in black and grey as a reference (see Fig. 4 of the main paper), and coloured lines correspond to alternatives tested in sensitivity analyses.

# 6 Stan code

All the models described in section 1 were implemented within Stan 2.15.1.

## 6.1 Model for $\mathcal{D}1$

```
data {
  // data
  int W; // number of records
  int O_t[W]; // number of reported cases
  int sumO_t[W]; // cumulative number of reported cases
  int pop; // island total population
  int siL; // length of discretized serial interval distribution (in weeks)
  vector[siL] siW; // discretized serial interval distribution

  // prior settings
  int P_R0_type; // 1=exponential; 2=uniform; 3=normal; 4=gamma; 0=hyperprior
  int P_rho_type; // 1=beta; 2=uniform; 0=hyperprior
  real<lower=0> P_R0[2];
  real<lower=0> P_rho[2];

  // hyperprior settings (only used for region-specific priors, type=0)
  real P_mu_R0[2]; // R0 mean (gamma distribution)
  real P_sigma_R0[2]; // R0 standard deviation (gamma distribution)
  real P_IL_mu_rho[2]; // rho mean (inverse logit scale, normal distribution)
  real P_IL_sigma_rho[2]; // rho standard deviation (inverse logit scale, gamma distribution)

  // prediction
  int pW; // number of weeks of prediction
}

parameters {
  // declaring island-specific random parameters
  real<lower=0> R0; // island-level base transmission
  real raw_rho; // reporting rate

  // declaring region-specific hyperparameters (only used for region-specific priors, type=0)
  real<lower=0> mu_R0;
  real<lower=0> sigma_R0;
  real IL_mu_rho;
  real<lower=0> IL_sigma_rho;

  // declaring dispersion parameter
  real<lower=0> phi;
}

transformed parameters {
  // declaring model intermediates
  real<lower=0,upper=1> rho; // rescaled raw_rho if region-specific prior
  real<lower=0> lp[W]; // mean prediction
  real<lower=0> sampledisp[W]; // dispersion = mean/phi
  vector[siL] lagO; // past reported cases
  real Ostar_t; // exposition
```

```
  if(P_rho_type==0) rho = inv_logit(raw_rho); else rho = raw_rho;
  for(i in 1:W) {
    // computing exposition from past reported cases
    if(i>1) {
      for(j in 1:siL) {
        if((i-j)>1) {
          lagO[j] = O_t[i-j];
        } else {
          lagO[j] = 0;
        }
      }
      Ostar_t = sum(lagO .* siW);
    } else {
      Ostar_t = 0;
    }
    // building negative binomial model
    lp[i] = R0 * Ostar_t * ( 1 - sumO_t[i] / ( rho * pop ) );
    if(lp[i]==0) lp[i] = 0.0001;
    sampledisp[i] = lp[i]/phi;
  }
}

model {
  // region-specific hyperpriors (only used for region-specific prior)
  mu_R0 ~ gamma(P_mu_R0[1],P_mu_R0[2]);
  sigma_R0 ~ gamma(P_sigma_R0[1],P_sigma_R0[2]);
  IL_mu_rho ~ normal(P_IL_mu_rho[1],P_IL_mu_rho[2]);
  IL_sigma_rho ~ gamma(P_IL_sigma_rho[1],P_IL_sigma_rho[2]);

  // island-specific prior for R0
  if(P_R0_type==1) R0 ~ exponential(P_R0[1]);
  if(P_R0_type==2) R0 ~ uniform(P_R0[1],P_R0[2]);
  if(P_R0_type==3) R0 ~ normal(P_R0[1],P_R0[2]);
  if(P_R0_type==4) R0 ~ gamma(P_R0[1],P_R0[2]);
  if(P_R0_type==0) R0 ~ normal(mu_R0,sigma_R0);

  // island-specific prior for rho
  if(P_rho_type==1) raw_rho ~ beta(P_rho[1],P_rho[2]);
  if(P_rho_type==2) raw_rho ~ uniform(P_rho[1],P_rho[2]);
  if(P_rho_type==0) raw_rho ~ normal(IL_mu_rho,IL_sigma_rho);

  // prior for phi
  phi ~ cauchy(0,2.5);

  // likelihood
  target += neg_binomial_2_lpmf(O_t|lp,sampledisp);
}

generated quantities {
  real log_lik[W]; // save the likelihood
  real pred_lp[W]; // predicted values
  real resid_lp[W]; // residuals
  real totlp;
  real totoverall;
```

```
  real attackrate;
  real pS;
  vector[siL] lagpO;
  real pOstar;
  real pO[pW];
  real plp;
  real ptotlp;
  real ptotoverall;
  real pattackrate;

  // diagnostics
  for (i in 1:W) {
    log_lik[i] = neg_binomial_2_lpmf(O_t[i]|lp[i],sampledisp[i]);
    pred_lp[i] = neg_binomial_2_rng(lp[i],sampledisp[i]);
    resid_lp[i] = O_t[i] - pred_lp[i];
  }

  // transformed outcomes
  totlp = sum(lp);
  totoverall = totlp / rho;
  attackrate = totoverall / pop;

  // forecasts
  pS = pop - totlp / rho ;
  lagpO = tail(to_vector(O_t),siL);
  for(week in 1:pW) {
    pOstar = 0;
    for (j in 1:siL) {
      pOstar = pOstar + (lagpO[siL+1-j] * siW[j]);
    }
    plp = R0 * pOstar * pS / pop;
    pO[week] = 0;
    if(plp>0) {
      pO[week] = neg_binomial_2_rng(plp,plp/phi);
    }
    pS = pS - ( pO[week] / rho );
    for(j in 1:(siL-1)) {
      lagpO[j] = lagpO[j+1];
    }
    lagpO[siL] = pO[week];
  }
  ptotlp = sum(pO) + totlp;
  ptotoverall = ptotlp / rho;
  pattackrate = ptotoverall / pop ;
}
```

### 6.2 Model for $\mathcal{D}2$

```
data {
  int W; // number of records
  int K; // number of islands
  int O_t[W]; // number of reported cases
```

```
  real Ostar[W]; // exposition (past incidence weighted by the discretized serial interval)
  int sumO_t[W]; // cumulative number of reported cases
  int island[W]; // island index (1 to K)
  int pop[K]; // island population
}

parameters {
  // declaring hyperparameters (normally distributed random effects)
  real<lower=0> mu_R0_CHIKV; // non-centered
  real<lower=0> sigma_R0_CHIKV;
  real IL_mu_rho_CHIKV; // non-centered, with logit transformation
  real<lower=0> IL_sigma_rho_CHIKV;

  // declaring random parameters by island (non-centered)
  real R0_CHIKV_TILDE[K]; // island-level base transmission for CHIKV
  real IL_rho_CHIKV_TILDE[K]; // reporting rate by island (inverse-logit transformed)

  // declaring dispersion parameter
  real<lower=0> phi;
}

transformed parameters {
  // declaring rescaled random parameters
  real<lower=0> R0_CHIKV[K]; // island-level base transmission for CHIKV
  real<lower=0,upper=1> rho_CHIKV[K]; // reporting rate by island

  // declaring model intermediates
  real<lower=0> lp[W]; // mean prediction
  real<lower=0> sampledisp[W]; // dispersion = mean/phi

  // rescaling random parameters
  for(i in 1:K) {
    R0_CHIKV[i] = mu_R0_CHIKV + sigma_R0_CHIKV * R0_CHIKV_TILDE[i];
    rho_CHIKV[i] = inv_logit(IL_mu_rho_CHIKV + IL_sigma_rho_CHIKV * IL_rho_CHIKV_TILDE[i]);
  }

  // building negative binomial model
  for(i in 1:W) {
    lp[i] = R0_CHIKV[island[i]] * Ostar[i] * ( 1 - sumO_t[i] / ( rho_CHIKV[island[i]] * pop[island[i]] )[illegible]
    if(lp[i]==0) lp[i] = 0.0001;
    sampledisp[i] = lp[i]/phi;
    if(sampledisp[i]==0) sampledisp[i] = 0.0001;
  }
}

model {
  // priors
  mu_R0_CHIKV ~ gamma(1,0.2); // implies: R0 strictly positive, away from 0, probably between 1 and 5
  sigma_R0_CHIKV ~ cauchy(0,2.5);
  IL_mu_rho_CHIKV ~ normal(0,1.5); // implies: mu_rho_CHIKV uniform on [0,1]
  IL_sigma_rho_CHIKV ~ cauchy(0,2.5);
  for(i in 1:K) {
    R0_CHIKV_TILDE[i] ~ normal(0,1); // implies R0_CHIKV ~ normal( mu_R0_CHIKV,sigma_R0_CHIKV )
    IL_rho_CHIKV_TILDE[i] ~ normal(0,1); // implies rho_CHIKV ~ inverse_logit( normal( IL_mu_rho_CHIKV,[illegible]
```

```
  }
  phi ~ cauchy(0,2.5);

  // likelihood
  target += neg_binomial_2_lpmf(O_t|lp,sampledisp);
}

generated quantities {
  real log_lik[W]; // save the likelihood
  real pred_lp[W]; // predicted values around lp
  real resid_lp[W]; // residuals
  real mu_rho_CHIKV;

  // diagnostics
  for (i in 1:W) {
    log_lik[i] = neg_binomial_2_lpmf(O_t[i]|lp[i],sampledisp[i]);
    pred_lp[i] = neg_binomial_2_rng(lp[i],sampledisp[i]);
    resid_lp[i] = O_t[i] - lp[i];
  }

  // rescaling hyperparameter
  mu_rho_CHIKV = inv_logit(IL_mu_rho_CHIKV);
}
```

### 6.3 Model for $\mathcal{D}3$

```
data {
  int W; // number of records
  int K; // number of islands
  int J; // number of viruses (=2)
  int O_t[W]; // number of reported cases
  real Ostar[W]; // exposition (past incidence weighted by the discretized serial interval)
  int sumO_t[W]; // cumulative number of reported cases
  int island[W]; // island index (1 to K)
  int virus[W]; // virus index (0=CHIKV ; 1=ZIKV)
  int pop[K]; // island population
}

parameters {
  // declaring hyperparameters (normally distributed random effects)
  real<lower=0> mu_R0_CHIKV; // non-centered
  real<lower=0> sigma_R0_CHIKV;
  real IL_mu_rho_CHIKV; // non-centered, with logit transformation
  real<lower=0> IL_sigma_rho_CHIKV;

  // declaring random parameters by island (non-centered)
  real R0_CHIKV_TILDE[K]; // island-level base transmission for CHIKV
  real IL_rho_CHIKV_TILDE[K]; // reporting rate by island (inverse-logit transformed)

  // declaring delta parameters between CHIKV and ZIKV
  real<lower=0> beta_R0;
  real<lower=0> beta_rho;
```

```
  // declaring dispersion parameter
  real<lower=0> phi;
}

transformed parameters {
  // declaring rescaled random parameters
  real<lower=0> R0_CHIKV[K]; // island-level base transmission for CHIKV
  real<lower=0,upper=1> rho_CHIKV[K]; // reporting rate by island

  // declaring ZIKV parameters in relation to CHIKV
  real<lower=0> R0_ZIKV[K];
  real<lower=0,upper=1> rho_ZIKV[K];

  // declaring model intermediates
  real<lower=0> lp[W]; // mean prediction
  real<lower=0> sampledisp[W]; // dispersion = mean/phi

  // rescaling random parameters
  for(i in 1:K) {
    R0_CHIKV[i] = mu_R0_CHIKV + sigma_R0_CHIKV * R0_CHIKV_TILDE[i];
    R0_ZIKV[i] = R0_CHIKV[i] * beta_R0;
    rho_CHIKV[i] = inv_logit(IL_mu_rho_CHIKV + IL_sigma_rho_CHIKV * IL_rho_CHIKV_TILDE[i]);
    rho_ZIKV[i] = rho_CHIKV[i] * beta_rho;
  }

  // building negative binomial model
  for(i in 1:W) {
    lp[i] = ( virus[i] == 0 ? R0_CHIKV[island[i]] : R0_ZIKV[island[i]] ) * Ostar[i] *
        ( 1 - sumO_t[i] / ( ( virus[i] == 0 ? rho_CHIKV[island[i]] : rho_ZIKV[island[i]] ) * pop[island[
    if(lp[i]==0) lp[i] = 0.0001;
    sampledisp[i] = lp[i]/phi;
    if(sampledisp[i]==0) sampledisp[i] = 0.0001;
  }
}

model {
  // priors
  mu_R0_CHIKV ~ gamma(1,0.1); // implies: R0 strictly positive, away from 0, probably between 1 and 10
  sigma_R0_CHIKV ~ cauchy(0,2.5);
  IL_mu_rho_CHIKV ~ normal(0,1.5); // implies: mu_rho_CHIKV uniform on [0,1]
  IL_sigma_rho_CHIKV ~ cauchy(0,2.5);
  beta_R0 ~ exponential(1);
  beta_rho ~ exponential(1);
  for(i in 1:K) {
    R0_CHIKV_TILDE[i] ~ normal(0,1); // implies R0_CHIKV ~ normal( mu_R0_CHIKV,sigma_R0_CHIKV )
    IL_rho_CHIKV_TILDE[i] ~ normal(0,1); // implies rho_CHIKV ~ inverse_logit( normal( IL_mu_rho_CHIKV,I
  }
  phi ~ cauchy(0,2.5);

  // likelihood
  target += neg_binomial_2_lpmf(O_t|lp,sampledisp);
}
```

```
generated quantities {
  real log_lik[W]; // save the likelihood
  real pred_lp[W]; // predicted values around lp
  real resid_lp[W]; // residuals
  real mu_rho_CHIKV;
  real IL_mu_rho_ZIKV;
  real mu_R0_ZIKV;
  real mu_rho_ZIKV;

  // diagnostics
  for (i in 1:W) {
    log_lik[i] = neg_binomial_2_lpmf(O_t[i]|lp[i],sampledisp[i]);
    pred_lp[i] = neg_binomial_2_rng(lp[i],sampledisp[i]);
    resid_lp[i] = O_t[i] - lp[i];
  }

  // rescaling hyperparameter
  mu_rho_CHIKV = inv_logit(IL_mu_rho_CHIKV);
  mu_rho_ZIKV = mu_rho_CHIKV * beta_rho;
  IL_mu_rho_ZIKV = logit(mu_rho_ZIKV);
  mu_R0_ZIKV = mu_R0_CHIKV * beta_R0;
}
```

## 7 References
