# Supplementary figures and images for: Improving early epidemiological assessment of emerging Aedes-transmitted epidemics using historical data
Source: PLoS Negl Trop Dis. 2018 Jun 4;12(6):e0006526. doi: 10.1371/journal.pntd.0006526 (PMC6002135; doi:10.1371/journal.pntd.0006526)

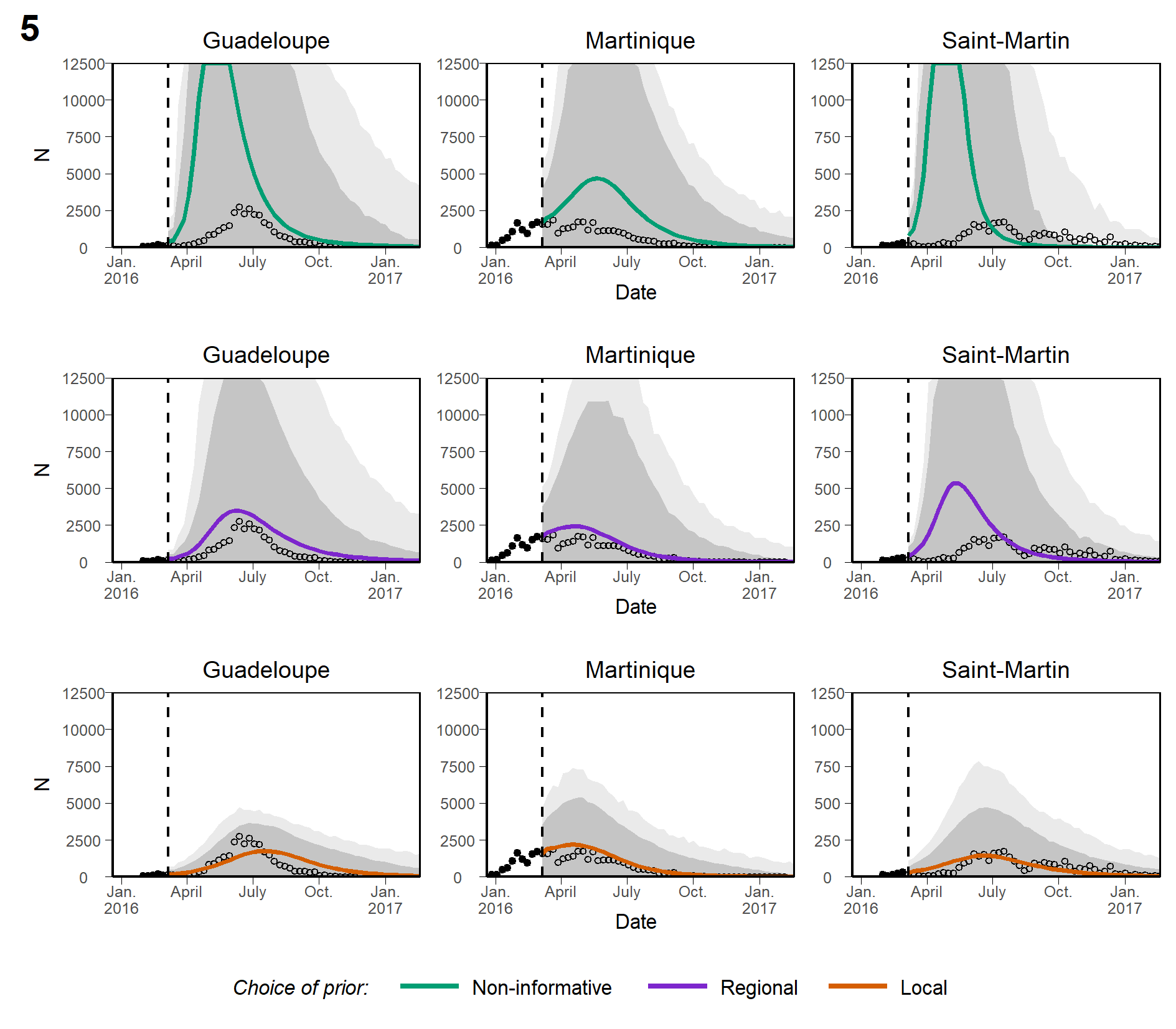

Supplement: S1 Fig — (GIF) [file pntd.0006526.s002.gif]
